# Mast cells and the development of allergic airway disease

**DOI:** 10.1186/1745-6673-3-S1-S2

**Published:** 2008-02-27

**Authors:** Sebastian Reuter, Christian Taube

**Affiliations:** 1 III. Medical Clinic, Dept. of Pulmonary Medicine, Johannes-Gutenberg-University, Langenbeckstr. 1, 55101 Mainz, Germany

## Abstract

Murine models have highlighted the importance of T-cells and T_H_2 cytokines in development of allergen-induced airway disease. In contrast, the role of mast cells for the development of allergic airway disease has been controversial. Recent studies in murine models demonstrate a significant contribution of mast cells during the development of airway hyperresponsiveness and airway inflammation. Furthermore these models have allowed identifying certain mast cell-produced mediators (e.g. histamine and leukotriene B4) to be involved in the recruitment of effector T-cells into the lung. Additionally, mast cell-produced TNF can directly activate T_H_2 cells and contribute to the development of allergic airway disease. These new findings demonstrate a complex role of mast cells and their mediators, not only as effector cells, but also during sensitization and development of allergic airway disease. Therefore mast cells and certain mast cell-produced mediators might be an interesting target for the prevention and treatment of allergic asthma.

## Introduction

For decades mast cells have been known as important effector cells for adaptive immune responses. Functions of mast cells include both IgE-dependent mucosal immunity to parasites but also dysregulated allergic responses to environmental antigens. In patients with allergic asthma, allergen-specific activation of mast cells through IgE bound to the high-affinity IgE receptor (FcΕRI) induces early airway responses following allergen exposure. Also increased numbers of mast cells are found in close proximity to airway smooth muscle in patients with allergic asthma, suggesting their potential role in the development and maintenance of allergic airway disease [1].

Murine models have helped to gain insights into the pathogenesis of airway inflammation and airway responsiveness [2]. These models have highlighted the importance of T-cells and T-cell mediated cytokines for the development of the disease. Mast cell-deficient mice can be utilized to investigate the role of mast cells in these models. Most commonly two mast cell-deficient mouse strains, the WBBF1-Kit^W^/Kit^W-v^ and more recently the C57BL/6-Kit^W-sh^/Kit^W-sh^ are used [3]. Both strains are mast cells-deficient but also lack melanocytes. Additionally, the WBBF1-Kit^W^/Kit^W-v^ mice are sterile, anaemic, and lack intestinal cells of Cajal, whereas the C57BL/6-Kit^W-sh^/Kit^W-sh^ are not sterile or anaemic [3].

## Mast cells contribute to the development of allergic airway disease

The role of mast cells for the development of allergic airway disease in murine models is critically dependent on the sensitization and allergen exposure protocol. In studies using systemic sensitization with adjuvant hyperresponsiveness (AHR) and airway inflammation are similar in mast cell- or IgE-deficient mice compared to wild-type mice [4-6]. However, in models with less potent sensitization protocols, a role of mast cells for the induction of non-allergic [7] but also allergen-induced airway disease [8-10] can be demonstrated. In these models the presence of mast cells in already sensitized animals is essential for the initiation of airway inflammation and AHR. Further studies, using mast cell reconstitution protocols, revealed that the expression of the Fc receptor γ chain in mast cells [10], necessary for the surface expression of the Fcγ receptors I and III and the FcεRI receptor is pivotal for the induction of most features of allergen-induced lung pathology. In studies using an inhalation allergen exposure, development of increased airway reactivity was associated with the expression of FcεRI on mast cells [11]. These results underscore that mast cells are involved in the development of an allergic airway disease in already sensitized hosts.

Mast cells not only play an important role during the effector phase of allergic airway disease but also could be involved during sensitization to an allergen. Intranasal exposure with allergen alone does not induce a specific T-cell response. In contrast, exposure with allergen in combination with a low dose of bacterial lipopolysaccharides (LPS) induces a specific T-cell response [12] and consecutive challenge with the allergen then leads to eosinophilic airway inflammation. Interestingly, mast cell-deficient mice exposed intranasally with allergen and low dose LPS do not develop eosinophilic airway inflammation following rechallenge. However, mast cell-deficient mice reconstituted with wild-type mast cells developed airway inflammation similar to wild-type animals, suggesting an involvement of mast cells during the sensitization process [13]. Indeed, mast cells express several Toll like receptors (TLR) including TLR-4 on their surface and release cytokines and chemokines following exposure to TLR-ligands [14] and mast cell-deficient mice reconstituted with TLR-4 deficient mast cells, did not develop airway eosinophilia following allergen exposure [13]. These findings suggest that IgE-independent mast cell activation through TLR-4 is necessary for sensitization to an allergen administered directly into the lung, further extending the function and role of mast cells in the development of allergic diseases.

## Mast cell-produced mediators

Mast cells, following activation, degranulate and produce a plethora of lipid mediators, cytokines, and chemokines [3]. Several of these mediators might contribute through different mechanisms to the development of AHR. One potential mechanism could be the secretion of cytokines and especially IL-13 [15]. IL-13 is capable to directly induce AHR and blockade of IL-13 has been shown to be an effective treatment approach in acute [16,17] and chronic [18] models of allergic airway disease. However, IL-13 deficient mast cells, when transferred into FcεRI-receptor deficient mice, where still able to induce increased airway reactivity. This finding suggests that IL-13 production from mast cells does not play a primary role in the development of allergic airway disease [11].

Other mechanisms could be the secretion of chemo-attractants and chemokines, which lead to the recruitment of other cell populations, such as effector T-cell into the lung and airways. Recently an interaction with mast cells has been described for allergen specific CD8-positive T-effector cells. These *in vitro* studies have shown that migration of CD8-positive T-effector cells can be initiated by mast cell activation. Furthermore T-cell migration was regulated by leukotriene B4 (LTB4) produced by mast cells and the expression of the receptor 1 (BLT-1) on the T-cells [19]. CD8-positive T-cells [20,21] and especially allergen experienced CD8-positive T effector cells (Teff) are critical for the development of AHR and airway inflammation [22]. Following sensitization and challenge increased migration of these cells into the lung can be demonstrated [22] and this migration is dependent on LTB4 [23]. The contribution of mast cell produced LTB4 for Teff cell recruitment was assessed in a previously described model of passive sensitization [24]. Mice were passively sensitized with OVA-specific IgE. Mast cell-, FcεRI- and CD8-deficient mice showed only low airway reactivity following passive sensitization compared to passively sensitized wild-type mice. Increased airway reactivity could be reconstituted in CD8-deficient mice by adoptive transfer of allergen-specific Teff cells. Only transferred BLT-1 expressing Teff migrated effectively to the lung and reconstituted AHR whereas BLT-1 deficient Teff failed to do so [25]. A potential contribution of basophils in this response can not be excluded as basophils express the FcΕRI in the mouse [26].

Also other mast cell-produced mediators are of interest. Histamine receptor 1 has recently been shown to be important for the development of allergic airway disease and also for T-cell trafficking [27]. Another important mast cell-produced mediator is Tumor Necrosis Factor (TNF). In other disease models mast cell-derived TNF is important for the induction and promotion of initial inflammatory events [28-33]. Several studies have also demonstrated a contribution of TNF to the induction of allergic airway diseases [34-36]. Sensitized mast cell-deficient mice express lower levels of TNF in BAL fluid compared to sensitized and challenged wild-type mice and reconstitution with bone marrow-derived mast cells restored BAL fluid levels of TNF and AHR [37]. In a recent study Nakae *et al*. directly evaluated the role of mast cell-produced TNF for the induction of allergic airway disease using a mast cell reconstitution model. Similar to previous studies, mast cell-deficient animals fail to develop allergic airway disease whereas mice reconstituted with mast cells derived from wild-type mice developed allergic airway disease. In contrast, mice reconstituted with mast cells derived from TNF-deficient donors have less airway inflammation and AHR, demonstrating that mast cell-produced TNF is pivotal for the induction of allergic airway disease [38]. Furthermore the authors point out an enhanced proliferation of T_H_2 cells induced by mast cell-produced TNF, suggesting that mast cells induce activation of allergen specific T-cells in the sites of inflammation. These findings are of clinical interest as recent studies in patients with moderate [39] and also more severe asthma [40,41] described beneficial effects of treatment with TNF neutralizing antibodies on lung function and airway reactivity.

## Conclusion

Recent studies have outlined a critical role of mast cells for the development of acute allergic airway disease, especially in sensitization protocols without an adjuvant. Furthermore there is evidence that mast cells are also involved during the sensitization to inhaled allergens by IgE-independent activation through TLR. Still little is known about the role of the different mediators produced and secreted by mast cells. Several mast cell-produced mediators like LTB4, histamine and TNF have been identified to contribute to the development of allergic airway disease. Further research is needed as increasing knowledge about these pathways might allow a more targeted therapy of allergic asthma.

## Authors' contributions

S.R. designed and wrote the manuscript, C.T. designed and wrote the manuscript

## Competing interests

The authors declare that they have no competing interests.
